# A Prospective Surveillance Study of Candidaemia: Epidemiology, Risk Factors, Antifungal Treatment and Outcome in Hospitalized Patients

**DOI:** 10.3389/fmicb.2016.00915

**Published:** 2016-06-16

**Authors:** Ranjith Rajendran, Leighann Sherry, Ashutosh Deshpande, Elizabeth M. Johnson, Mary F. Hanson, Craig Williams, Carol A. Munro, Brian L. Jones, Gordon Ramage

**Affiliations:** ^1^School of Medicine, College of Medical, Veterinary and Life Sciences, University of GlasgowGlasgow, UK; ^2^Microbiology Department, Glasgow Royal InfirmaryGlasgow, UK; ^3^Public Health England, Southwest LaboratoryBristol, UK; ^4^Microbiology Department, NHS LothianEdinburgh, UK; ^5^University of the West of ScotlandGlasgow, UK; ^6^Aberdeen Fungal Group, University of AberdeenAberdeen, UK

**Keywords:** *Candida albicans*, *Candida glabrata*, candidaemia, antifungals, drug resistance

## Abstract

This study provide an up-to-date overview of the epidemiology and risk factors for *Candida* bloodstream infection in Scotland in 2012/2013, and the antifungal susceptibility of isolates from blood cultures from 11 National Health Service boards within Scotland. *Candida* isolates were identified by chromogenic agar and confirmed by MALDI–TOF methods. Survival and associated risk factors for patients stratified as *albicans* and non-*albicans* cases were assessed. Information on the spectrum of antifungals used was collected and summarized. The isolates sensitivity to different antifungals was tested by broth microdilution method and interpreted according to CLSI/EUCAST guidelines. Forty one percent of candidaemia cases were associated with *Candida albicans*, followed by *C. glabrata* (35%), *C. parapsilosis* (11.5%), and remainder with other *Candida* spp. *C. albicans* and C. *glabrata* infections were associated with 20.9 and 16.3% mortality, respectively. Survival of patients with *C. albicans* was significantly lower compared to non-*C. albicans* and catheter line removal in *C. albicans* patients significantly increases the survival days. Predisposing factors such as total parenteral nutrition, and number of days on mechanical ventilation or in intensive care, were significantly associated with *C. albicans* infections. Fluconazole was used extensively (64.5%) for treating candidaemia cases followed by echinocandins (33.8%). Based on CLSI breakpoints, MIC test found no resistance to any antifungals tested except 5.26% fluconazole resistance among *C. glabrata* isolates. Moreover, by comparing to EUCAST breakpoints we found 3.95% of *C. glabrata* isolates were resistant to anidulafungin. We have observed a shift in *Candida* spp. with an increasing isolation of *C. glabrata*. Delay and choice of antifungal treatment are associated with poor clinical outcomes.

## Introduction

*Candida* species remain a significant cause of nosocomial bloodstream infections (BSIs), associated with prolonged hospital stay in the ICU and high healthcare cost (Rentz et al., 1998; Gudlaugsson et al., 2003). The global prevalence of candidemia was reported to be highly variable among different countries ranging between 1.1 and 14.4 cases per 10^5^ population (Odds et al., 2007; Arendrup, 2010; Gamaletsou et al., 2013; Quindos, 2014; Cleveland et al., 2015; Osmanov and Denning, 2015). The maximum of 14.4 cases per 10^5^ population was reported in USA (Cleveland et al., 2015), though in Scotland was recently reported to be 4.8 (Odds et al., 2007).

Reports in recent years have established various risk factors for *Candida* BSIs. The risk factors including broad-spectrum antibiotics, parenteral nutrition, immuno-suppression due to chemotherapy and radiotherapy, disruption of mucosal barriers due to surgery, ICU admission and indwelling medical devices such as central venous catheters (CVCs), are among the most important predisposing factors for *Candida* BSI (Dimopoulos et al., 2008; Leroy et al., 2009). Indeed, biofilm formation by *Candida spp.* and choice of antifungal has been shown to be risk factors for morbidity and mortality in candidaemia cases (Tumbarello et al., 2007, 2012; Rajendran et al., 2015). Though *Candida albicans* is the most prevalent, pathogenic and robust biofilm former of the *Candida* species, there are recent studies reporting non-*C. albicans* species (NCAS) as frequent causes of candidemia (Nguyen et al., 1996; Dimopoulos et al., 2008; Hachem et al., 2008). Prior patient exposure to antifungals, particularly to azoles, appears to be a predictor for NCAS associated candidaemia (Nguyen et al., 1996).

Catheters are commonly used in ICU patients (∼90%), and represent an easy entry route for *Candida* spp (Almirante et al., 2005). Biofilm formation by *Candida* spp. including *C. albicans* in indwelling lines is considerably difficult to treat with antifungals due to their intrinsic drug resistance (Ramage et al., 2012). Appropriate antifungal medication and catheter removal are critical in preventing patient mortality with line infections (Mermel et al., 2009; Cornely et al., 2012; Koehler et al., 2014). Delaying antifungal therapy appears to be a common practice, and the impact on mortality may be significant with catheter related infections. Fluconazole is a mainstay of treatment for candida BSI, however, they are not effective against *C. glabrata* or preformed biofilms (Lee et al., 2009; Ramage et al., 2012; Castanheira et al., 2016; Goncalves et al., 2016). In appropriate antifungal usage was reported to be associated with high morbidity and excess hospital cost (Zilberberg et al., 2010).

National epidemiological surveillance and antifungal sensitivity testing of *Candida* BSIs is vital to stratify high-risk patients and to identify the pattern of causative *Candida* spp., which will in turn formulate guidelines for management of candidaemia. Therefore the purpose of this study was to investigate this in Scotland.

## Materials and Methods

### Patients and Variables

A prospective study of all cases of candidaemia was carried out within Scotland under NHS Caldicott Guardian approval from March 2012 to February 2013. This work formed an audit and as such ethics approval and patient consent are not required in the NHS. This was mandated for the NHS by Health Service Circular: HSC 1999/012. *Candida* BSI was reported in 217 patients from 11 different health boards; clinical data was obtained from 150 patients. The complete data sets of patient demographics, ICU admissions, underlying medical conditions, information on immunosuppression, and details of antifungal therapy were collected through a review of the medical case notes from 8 health boards. Where available, patient outcomes were followed prospectively for 30 days or until death from first blood culture positive, and clinical details including the presence of indwelling medical devices in the 30 days prior to the occurrence of candidemia were also collected.

### Isolates and Antifungal Testing

Blood cultures were performed according to routine standard operating procedures in each of the referring laboratories. All clinical isolates were obtained at the beginning of episode 1 and independently identified using Colorex*Candida* chromogenic plates (E&O Laboratories Ltd, Bonnybridge, UK) and were stored in Microbank^®^ vials (Pro-Lab Diagnostics, Cheshire, UK) at -80°C until further use. Isolates identification were further confirmed from sub-culture on glucose-peptone plates by MALDI-TOF MS analysis using a Bruker Microflex to produce the spectra which were then analyzed using the Bruker database. MIC test for different antifungals were performed centrally at Mycology Reference Laboratory, Public Health England, Bristol, according to standard Clinical and Laboratory Standards Institute (CLSI) broth microdilution methodology (CLSI, 2008). Interpretive criteria used for fluconazole (FLZ), voriconazole (VRZ), and anidulafungin (ANID) were performed in accordance with the current CLSI guidelines (CLSI, 2012).

### Statistical Analysis

Initially, all data were numerically coded and labeled for each variable, which was analyzed using SPSS software (SPSS Inc., Chicago, IL, USA). Categorical variables were compared between groups using the two-tailed *χ*^2^ test or Fisher’s exact test, as appropriate. Two groups of any continuous variables were compared using Student’s *t*-test or Mann–Whitney *U*-test as appropriate. Variables showing a significant association with survival found by Student’s t test or *χ*^2^ test (**Supplementary Table S2**) were included into subsequent multivariate Cox’s regression analysis, to generate the survival curves.

## Results and Discussion

We undertook a retrospective analysis of candidaemia patients in Scotland as means of understanding the national epidemiology of *Candida* species, associated risk factors and isolates sensitivity to antifungal agents. Data from the most recent 2011 census list the population of Scotland at 5,295,403^1^ Therefore the population-based incidence of candidaemia in Scotland was calculated as 4.1 per 100,000 population for the year 2012/13. This data is comparable to those of 2005/06 (4.8 cases per 100,000 of population per year) (Odds et al., 2007). The minor discrepancy between these two studies could be explained by a growing population and from reporting of the false positive cases, and is therefore likely to represent a minimum estimate of the incidence of candidaemia in Scotland. There were approximately equal numbers of male and female patients with candidaemia and at a mean age of 63 years (standard deviation ± 20.3 years). During the study period, 33 out of 141 candidemia patients were admitted to the ICU (23.4%).

### Prevalence and Mortality Associated with *Candida* spp

The prevalence of the different *Candida* species isolated from the BS of candidaemia patients was assessed. Out of the 280 isolates collected in this study *C. albicans* predominated (41%), followed by *C. glabrata* (35%), *C. parapsilosis* (11.5%), *C. tropicalis* (3.6%), *C. lusitaniae* (3.6%), and other species (5.3%). Our previous Scottish candidaemia study (2005/06)(Odds et al., 2007) reported a prevalence of *C. albicans and C. glabrata* as 50 and 21%, respectively (**Supplementary Table S1**). This shows a changing trend in the prevalence of *Candida* spp in causing BSI, with a considerable decrease of *C. albicans* by 9% followed by a reciprocal increase of *C. glabrata* by 14%. *C. parapsilosis* did not show any clear change (∼12% in both studies). These fluctuations are in accordance with the global epidemiological changes, where in Denmark it has been shown that the overall prevalence of *C. albicans* has declined by 5% co-incidentally with an increase in *C. glabrata* by 6.9% (year 2010–2011 compared to 2004–2009) (Eggimann et al., 2011; Arendrup et al., 2013b). The reason for this is unclear; however, studies suggesting an increase in the use of FLZ have an impact on selection of *C. glabrata* (Pfaller and Diekema, 2007; Arendrup et al., 2013b). Of the 129 cases where patient mortality data was available, overall mortality was 41% (*n* = 53), which was primarily associated with *C. albicans* (49% [*n* = 26]), followed by *C. glabrata* (32% [*n* = 17]), *C. parapsilosis* (11.3% [*n* = 6]), and other species (7.7% [*n* = 4]).

### Therapy

Fluconazole therapy was the predominant antifungal treatment, out of 121 patients for which there was available antifungal usage data. We define the episode as a positive blood culture >21 days after the last positive culture or if a new *Candida* spp. isolated. In this study we have reported maximum three episodes from the isolate collection date up to 30 days time period unless they discharged early or died. In episode 1, 78 were treated with FLZ (64.5%), 17 with CAS (14%), 16 with ANID (13.2%), 8 with micafungin (6.6%), 1 with AmBisome (0.8%), and 1 with VRZ (1%) (**Table 1**). In episode 2 there was a 7.4% reduction in the use of FLZ, 8.3% reduction with CAS, 5.2% reduction with micafungin and 9.7% increase in the use of ANID compared to previous episode (**Table 1**). Out of 78 FLZ treated patients in episode 1 20 (25.6%) were switched to echinocandins in episode 2, due to resistance, line infection, no improvement or microbiological advice. Patients with *C. glabrata* showed the greatest treatment changes 37% (*n* = 10), though 50% (*n* = 5) of these patients’ clinical outcome was unsuccessful (dead). For *C. albicans* 20.7% (*n* = 6) of the patients’ treatment was changed, and only one was not successful. A previous study reported an association of inappropriate initial therapy such as FLZ to treat *C. glabrata*, which resulted in an increased length of hospital stay and excess hospital cost (Zilberberg et al., 2010). In addition, appropriate selection of initial antifungals by empirical use of echinocandins was reported to be even more cost effective than the use of FLZ (Zilberberg et al., 2009).

**Table 1:** Antifungals used.

		Fluconazole	Caspofungin	Anidulafungin	Micafungin	Ambisome	Voriconazole	Other
Episode^∗^ 1								
	No. (%)	78 (64.5)	17 (14)	16 (13.2)	8 (6.6)	1 (0.8)	1 (0.8)	0 (0)
	Patient ages (mean)	60.3	67.6	61.3	72.9	0.01	40	0 (0)
	Route	PO/IV	IV	IV	IV	IV	IV	
	No. days used (mean)	6.5	4.7	8.9	4.8	8	1	0(0)
	Outcome dead (no. (%))	20 (27.4)	7 (43.75)	8 (57.1)	2 (33.3)	1 (100)	0 (0)	0 (0)
	
Species specific [No. (%)]	*Candida albicans*	29 (59.2)	10 (20.4)	7 (14.3)	2 (4.1)	1 (2)	0 (0)	0 (0)


	*C. glabrata*	27 (69.2)	2 (5.1)	5 (12.8)	4 (10.3)	0 (0)	1 (2.6)	0 (0)
	*C. parapsilosis*	11 (55)	4 (20)	3 (15)	2 (10)	0 (0)	0 (0)	0 (0)
	*C. tropicalis*	5 (100)	0 (0)	0 (0)	0 (0)	0 (0)	0 (0)	0 (0)
	Others	6 (75)	1 (12.5)	1 (12.5)	0 (0)	0 (0)	0 (0)	0 (0)
	
Episode 2								
	No. (%)	40 (57.1)	4 (5.7)	16 (22.9)	1 (1.4)	2 (2.9)	1 (1.4)	6 (8.6)
	No. days used (mean)	9.7	8.3	8.5	19	16	22	7.7
	
Episode 3								
	No. (%)	13 (50)	1 (3.8)	1 (3.8)	1 (3.8)	2 (7.7)	7 (26.9)	1 (3.8)
	No. days used (mean)	7.8	19				10.83	2


*^∗^An episode is defined as a positive blood culture >21 days after the last positive culture or if a new *Candida* sp. isolated.*

**FIGURE 1:**
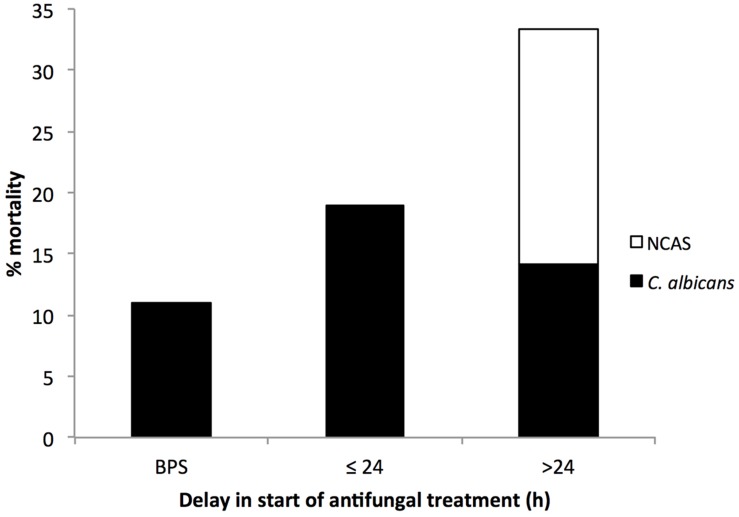
**Association between patient mortality and the timing of antifungal treatment.** The timing of antifungal therapy was determined to be before (BPS) or from the time when the first blood sample for different *Candida* culture positive was collected to the time when antifungal treatment was first administered to the patient. Number above each bar represents the overall % mortality over different antifungal timings and the black portion represent mortality associated with *Candida albicans* and white portion for NCAS infections.

Around 70% of candidemia patients do not receive empirical antifungal therapy within 24 h of the time the blood sample is drawn for culture. Previous studies have established the negative impact of delayed antifungal treatment on the clinical outcome of candidaemia cases (Morrell et al., 2005; Garey et al., 2006; Kollef et al., 2012). In our study, we found 9 (8.3%) patients received antifungal treatment before a positive sample (BPS) was collected, 21 (19.4%) patients received treatment between 0 and 24 h, and 78 (72%) patients after 24 h from the time at first positive blood sample for culture was drawn. The relationship between hospital mortality and the timing of the administration of antifungal treatment is shown in **Figure 1.** Patients receiving antifungal treatment BPS was drawn had a lower percentage mortality than patients begun on antifungal treatment between 1–24 h or >24 h (11% versus 19 or 33.1%). This is in line with a previous study that reported an association of delayed treatment by >12 h with increased hospital mortality (*p* < 0.05) (Morrell et al., 2005; Kollef et al., 2012). Of the patients with a BSI, we stratified groups according to the presence of *C. albicans* or NCAS, as previously described (Tumbarello et al., 2007; Dimopoulos et al., 2008). The data shows that delayed treatment has a considerable impact on NCAS associated mortality compared to *C. albicans*. Limitations of the present study include differences in clinical practice patterns across the different ward and different health boards, incomplete data sets and as well as information on antifungal dosing practices. Evading delays in treatment of candidemia patients is challenging due to use of culture based diagnosis methods.

### Comparison of *C. albicans* with NCAS Associated Candidaemia

**FIGURE 2:**
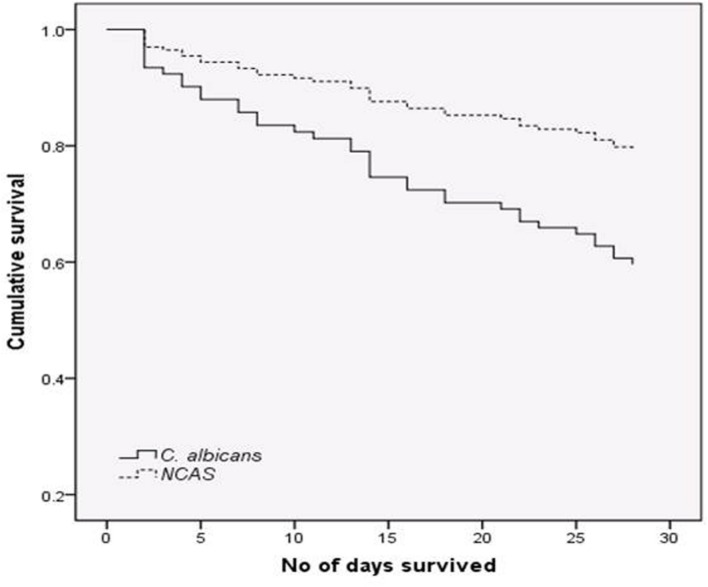
**Survival analysis for candidaemia patients.** Patients with bloodstream infections (BSIs) were grouped by *C. albicans* (n = 52) or non-*C. albicans* (NCAS) spp. (n = 72) and censored at death, or day 30. Cox-regression plots adjusted for patient age is shown. Comparison between these curves found a statistically significant difference in mortality rate (*p* < 0.05).

**FIGURE 3:**
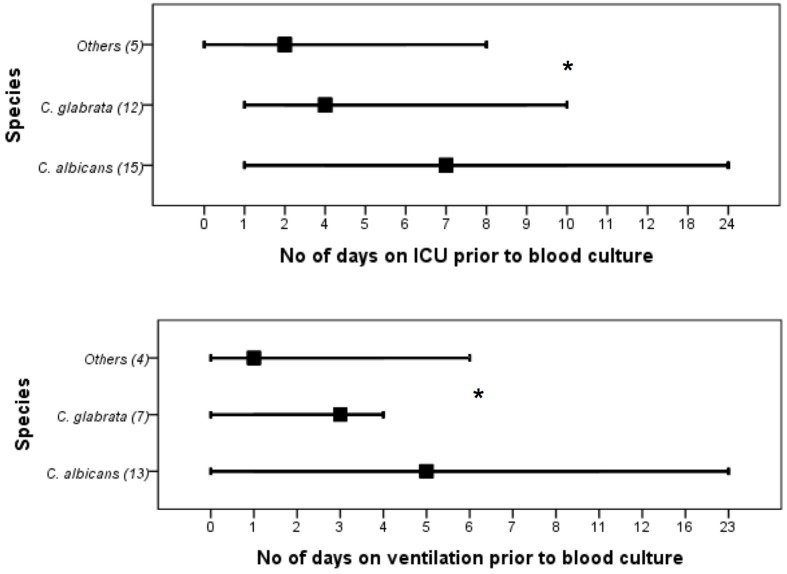
**Days in the intensive care unit (ICU) or days in the ventilation before the extended prevalence of *C. albicans*, *C. glabrata* or other *Candida* spp. infection.** Values in bracket indicate number of patients included in each group. ^∗^*C. albicans* vs NCAS (*p* < 0.05).

The proportion of *C. albicans* and NCAS associated infections was considerable among candidaemia cases from different health boards, i.e., Ayrshire and Arran ([*n* = 22] 50 and 50%), Fife ([*n* = 11] 27.3 and 72.7%), Forth valley ([*n* = 11] 27.3 and 72.7%), Grampian ([*n* = 14] 50 and 50%), Greater Glasgow and Clyde ([*n* = 39] 38.5 and 61.5), Lanarkshire ([*n* = 9] 33.3 and 66.7%), Lothian ([*n* = 26] 42.3 and 57.7%), and Tayside ([*n* = 18] 38.9 and 61.1%). Overall, survival analysis found a significantly higher mortality with patients infected with *C. albicans* compared to NCAS (*p* < 0.05) (**Figure 2**). Though previous studies from US, Greece and Taiwan, compared the crude mortality of *C. albicans* with NCAS and showed mixed results, here we showed a significant differences between these spp. in patient survival curve analysis (Dimopoulos et al., 2008; Moran et al., 2009; Chi et al., 2011). The various risk factors were analyzed to find its influence on causing BSI by either *C*. *albicans* or NCAS (**Table 2**). Patient demographics, including age and sex were not statistically significant association with species. Factors such as patients received total parenteral nutrition; no of days on ICU and number of days on mechanical ventilation prior to blood culture were independently related to infection by *C. albicans* (*p* = 0.030, *p* = 0.045, and *p* = 0.035, respectively). The average number of days on ICU or ventilation BPS for different *Candida* spp. is displayed in **Figure 3.** Though other variables have potential impact on infections caused by different *Candida* spp. we have not found any statistically significant correlation in this study. A similar study from Italy showed a significant relation between solid organ cancer, immunosuppressive therapy, indwelling urinary catheter and type of fungal isolate from candidemia patients (Tumbarello et al., 2007). Furthermore, other risk factors such as neutropenia, glucocorticosteroids, parenteral nutrition and CVCs were also reported to be associated with *C. albicans* and NCAS infections (Dimopoulos et al., 2008).

**Table 2:** Comparison of risk factors for *C. albicans* and NCAS infections.

Variables	% (No. patients)		*p*
		
	*C. albicans*	NCAS^∗^	
Mean ages	61.8 (60)	63.8 (90)	0.548
Male sex	27 (60)	50 (90)	0.136
Diabetes	19 (59)	29 (90)	0.572
Surgery in 1 month^∗∗^	29 (56)	41 (85)	0.405
Radiotherapy	2 (13)	9 (22)	0.115
Chemotherapy	7 (13)	11 (23)	0.500
Solid organ transplant	2 (60)	3 (89)	0.680
Metastatic	12 (14)	13 (24)	0.077
Solid Tumor	17 (59)	36 (88)	0.093
Abdominal surgery	22 (27)	26 (31)	0.541
Autoimmune or genetic disorder	4(60)	9 (89)	0.338
Renal failure	20 (52)	30 (77)	0.552
Liver disease	5 (52)	9 (82)	0.522
ICU admission	15 (54)	18 (79)	0.325
**No of days on ventilation^∗∗^ (mean)**	**7.08 (13)**	**2.18(11)**	**0.035**
**No of days on ICU^∗∗^ (mean)**	**7.87 (15)**	**4.12 (17)**	**0.045**
Line *in situ*	53 (54)	76 (80)	0.328
**Total parenteral nutrition**	**27 (51)**	**27 (78)**	**0.030**
Antibiotics in 2 weeks ^∗∗^	49 (52)	69 (78)	0.213
Antifungals in 3 months ^∗∗^	9 (38)	17 (77)	0.417
Steroid in 3 months ^∗∗^	16 (56)	17 (82)	0.195


*^∗^NCAS, Non-*Candida albicans* species. ^∗∗^, prior to blood culture. Boldface indicates a significant result.*

**FIGURE 4:**
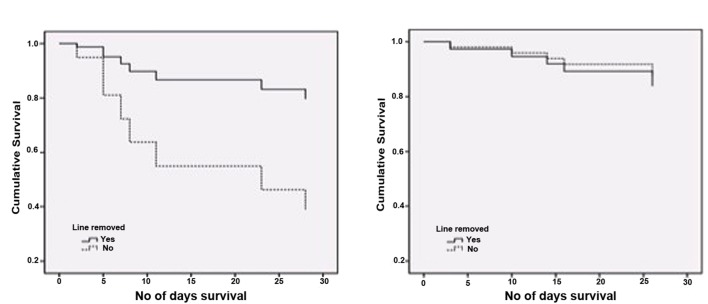
**Survival analysis for candidaemia patients with and without line removal.** Cox-regression plots adjusted for patient age is shown **(A)** Patients with *C. albicans* infection (*n* = 24); *p* < 0.05, **(B)** patients with non-*C. albicans* infections (NCAS [*n* = 37]). *p* > 0.05 for *C. albicans* vs NCAS.

In this study, out of 134 patient’s record, 129 were found to be inserted with either central or peripheral CVC. There was no significant difference in the rate of lines indwelling between *C. albicans* and NCAS groups. Within *C. albicans* and NCAS groups 18 and 29 patients, respectively, were line removed after diagnosis. Survival analysis shows a significantly higher survival of patients infected with *C. albicans* who have line removal compared to the non- removal (*p* < 0.05) (**Figure 4A**). Conversely, no significant difference was found with line removal in NCAS group (*p* > 0.05) (**Figure 4B**). The reason for this may be the high biofilm forming ability of *C. albicans* in catheters compared to NCAS such as *C. glabrata*. The biofilm phenotype as a significant clinical entity and this phenotype are highly recalcitrant to antifungal therapy (Rajendran et al., 2015). This data support the current guidelines for the management of catheter associated infection and their clinical management which indicate that where possible the catheter should be removed in non-neutropenic patients (Mermel et al., 2009; Cornely et al., 2012; Koehler et al., 2014). The caveat of this study includes the complicated nature of the patient population, frequency of concomitant bacterial infection and prior exposure to antimicrobials.

### Antifungal Sensitivity

A total of 204 clinical isolates were assessed for planktonic MIC testing of five different antifungal drugs. Concentration range, MIC_50_ and MIC_90_ for each antifungal tested against different *Candida* spp. are presented in **Table 2.** We applied interpretive breakpoints defined by CLSI for FLZ, VRZ, and ANID. No resistance was observed for *C. albicans*, *C. tropicalis*, or *C. parapsilosis* for any antifungal tested. However, the results showed a considerable prevalence of low azole susceptibility among *C. glabrata* isolates, for which was either intermediate (94.74%) or resistant (5.26%) to FLZ. Currently, no interpretable breakpoints for amphotericin B (AMB) and flucytosine (FLY) exist, therefore MIC values and the range of the measured MICs are given and no statement concerning the clinical resistance could be made (**Table 3**). Furthermore, comparing to EUCAST breakpoints (As of 2013, Arendrup et al., 2013a) we found that 3 (3.95%) of *C. glabrata* isolates were resistant to anidulafungin (**Table 3**). Our susceptibility data are similar to those of other studies published in recent years, with limited or no resistance found among *C. albicans, C. parapsilosis* or *C. tropicalis* isolates (Odds et al., 2007). Conversely all isolates of *C. glabrata* were considered intermediate for fluconazole except four resistance based on recent CLSI guidelines (CLSI, 2012). The decreased susceptibility of *C. glabrata* to the azoles is a well-known concern. Logically, the implications would suggest that patients with *C. glabrata* were at the greatest risk, though our data shows that mortality in *C. albicans* infection was significantly greater than *C. glabrata* infection. This provides further evidence that the biofilm phenotype as a significant clinical entity and this phenotype are highly recalcitrant to antifungal therapy (Ramage et al., 2012). Though we not found any resistance with *C. albicans* isolates in their planktonic form, with our expanded knowledge on biofilm susceptibility to antifungals, the use of these MIC values to treat biofilm based infections is a matter of concern (Kuhn et al., 2002; Nett et al., 2011; Rajendran et al., 2015).

**Table 3:** Clinical and Laboratory Standards Institute antifungal sensitivities of *Candida* spp.

Species (no. tested)	Agent	MIC (mg/L)				
				
		Range	MIC_50_^∗^	MIC_90_^∗∗^	No. I (%)	No. R (%)
*C. albicans* (*n* = 86)	AMB	<0.125–0.5	0.25	0.25		
	FLY	<0.125–0.5	<0.125	<0.125		
	FLZ	<0.125–8	<0.125	0.25	0	0
	VOR	<0.03–0.06	<0.03	0.06	0	0
	ANID	<0.015	<0.015	<0.015	0	0

*C. glabrata* (*n* = 76)	AMB	0.125–0.5	0.25	0.5		
	FLY	<0.125–2	<0.125	<0.125		
	FLZ	<0.125–>64	4	16	75 (94.74)	4(5.26)
	VOR	<0.03–4	0.06	0.25	0	0
	ANID	<0.015–0.25	0.03	0.03	0	0

*C. parapsilosis* (*n* = 29)	AMB	0.125–0.25	0.25	0.25		
	FLY	<0.125	<0.125	<0.125		
	FLZ	<0.125–16	0.5	1	0	0
	VOR	<0.03	<0.03	<0.03	0	0
	ANID	0.03–2	1	1	0	0

*C. tropicalis* (*n* = 12)	AMB	0.25–0.5	0.5	0.5		
	FLY	<0.125	<0.125	<0.125		
	FLZ	<0.125–0.5	0.25	0.5	0	0
	VOR	<0.03	<0.03	<0.03	0	0
	ANID	<0.015–0.06	0.03	0.03	0	0


*^∗^MIC_50_, Minimum inhibitory concentration for 50% of isolates tested. ^∗∗^MIC_90_, Minimum inhibitor concentration for 90% of isolates tested.*

In summary, the data herein shows a changing trend in the prevalence of NCAS causing BSI in Scotland, emphasizing the need for early and appropriate antifungal treatment for patients at high risk for candidaemia. In addition, removal of infected catheter lines, especially with *C. albicans*, have been shown to be associated with positive clinical outcome. This suggests the necessity for the development of rapid diagnosis methods for catheter related biofilm infections, which in turn as an aid to improving antifungal therapy.

## Author Contributions

RR, LS, AD, EJ, and MH participated in isolate and clinical data collection, study design, and carried out the experimental studies, performed statistical analysis, and were responsible for the manuscript. CW and CM participated in study design, statistical analysis, and helped draft the manuscript. GR and BJ conceived the study, participated in study design, data analysis and were responsible for writing and submission of the final manuscript. All authors read and approved the manuscript.

## Conflict of Interest Statement

The authors declare that the research was conducted in the absence of any commercial or financial relationships that could be construed as a potential conflict of interest.

## Footnotes

1 http://www.scrol.gov.uk/population-households.
