# Supplementary material for: A Prospective Surveillance Study of Candidaemia: Epidemiology, Risk Factors, Antifungal Treatment and Outcome in Hospitalized Patients
Source: Front Microbiol. 2016 Jun 16;7:915. doi: 10.3389/fmicb.2016.00915 (PMC4910670; doi:10.3389/fmicb.2016.00915)
Supplement: Supplementary file 1 [file Table_1.DOCX]

**Supplementary table 1 – Percentage *Candida* spp isolated from blood cultures from candidaemia patients in Scotland**

| **Species** | **Percentage isolates** | |
| --- | --- | --- |
|  | 2012/13*  (n=280) | 2005/06  (n=300) |
| *C. albicans* | 41 | 52 |
| *C. glabrata* | 35 | 22.7 |
| *C. parapsilosis* | 11.5 | 12 |
| *C. tropicalis* | 3.6 | 2 |
| *C. lusitaniae* | 3.6 | 2 |
| others | 5.3 | 9.6 |

* No. patients: 2012/13 - 217, 2005/06 - 242.
