# Supplementary material for: A Prospective Surveillance Study of Candidaemia: Epidemiology, Risk Factors, Antifungal Treatment and Outcome in Hospitalized Patients
Source: Front Microbiol. 2016 Jun 16;7:915. doi: 10.3389/fmicb.2016.00915 (PMC4910670; doi:10.3389/fmicb.2016.00915)
Supplement: Supplementary file 2 [file Table_2.DOCX]

| Variables |  | | p |
| --- | --- | --- | --- |
|  | % Survived | %Died |  |
| **Age (mean±SD)** | **60 ± 22** | **68 ± 16** | **0.022** |
| Male sex | 50.6 (44) | 54.5 (24) | 0.714 |
| Diabetes | 26.7 (23) | 34.1 (15) | 0.419 |
| Surgery | 48.2 (41) | 53.5 (23) | 0.708 |
| Radiotherapy | 38.9 (7) | 27.3 (3) | 0.694 |
| Chemotherapy | 58.8 (10) | 53.8 (7) | 1 |
| Solid organ transplant | 4.6 (4) | 0 (0) | 0.3 |
| Metastatic | 63.2 (12) | 81.8 (9) | 0.419 |
| Solid Tumour | 32.9 (28) | 38.6 (17) | 0.562 |
| Autoimmune or genetic disorder | 10.3 (9) | 4.7 (2) | 0.336 |
| Renal failure | 34.6 (28) | 42.9 (15) | 0.41 |
| Liver disease | 7.4 (6) | 17.9 (7) | 0.115 |
| Concomitant bacterial or viral infection | 78.8 (52) | 82.4 (28) | 0.795 |
| ICU admission | 20.2 (17) | 31 (13) | 0.191 |
| Parenteral nutrition | 40.5 (32) | 38.1 (16) | 0.847 |
| Antifungals in previous 3 months | 24 (18) | 14.6 (6) | 0.338 |
|  |  |  |  |

Supplementary Table 2 - Variables stratified according to the survival or death status at the 30-day endpoint

Values in brackets indicate the total no. of patients assessed for each variables.

Boldface indicates a significant result.
